# Natural plant diet impacts phenotypic expression of pyrethroid resistance in *Anopheles* mosquitoes

**DOI:** 10.1038/s41598-022-25681-6

**Published:** 2022-12-12

**Authors:** Prisca S. L. Paré, Domonbabele F. D. S. Hien, Koama Bayili, Rakiswendé S. Yerbanga, Anna Cohuet, David Carrasco, Edwige Guissou, Louis-Clément Gouagna, Koudraogo B. Yaméogo, Abdoulaye Diabaté, Rickard Ignell, Roch K. Dabiré, Thierry Lefèvre, Olivier Gnankiné

**Affiliations:** 1 Institut de Recherche en Sciences de la Santé (IRSS), Bobo-Dioulasso, Burkina Faso; 2 MIVEGEC, Université de Montpellier, IRD, CNRS, Montpellier, France; 3 Laboratoire d’Entomologie Fondamentale et Appliquée (LEFA), Unité de Formation et de Recherche - Sciences de la Vie et de la Terre (UFR-SVT), Université Joseph KI-ZERBO (UJKZ), Ouagadougou, Burkina Faso; 4 Laboratoire Mixte International sur les Vecteurs (LAMIVECT), Bobo-Dioulasso, Burkina Faso; 5 Institut des Sciences et Techniques (INSTech - BOBO), Bobo‑Dioulasso, Burkina Faso; 6 Department of Plant Protection Biology, Unit of Chemical Ecology, Disease Vector Group, Swedish University of Agricultural Sciences, Uppsala, Sweden

**Keywords:** Ecology, Evolution, Systems biology, Zoology

## Abstract

Success in reducing malaria transmission through vector control is threatened by insecticide resistance in mosquitoes. Although the proximal molecular mechanisms and genetic determinants involved are well documented, little is known about the influence of the environment on mosquito resistance to insecticides. The aim of this study was to assess the effect of plant sugar feeding on the response of *Anopheles gambiae *sensu lato to insecticides. Adults were fed with one of four treatments, namely a 5% glucose control solution, nectariferous flowers of *Barleria lupulina,* of *Cascabela thevetia* and a combination of both *B. lupulina* + *C. thevetia*. WHO tube tests were performed with 0.05% and 0.5% deltamethrin, and knockdown rate (KD) and the 24 h mosquito mortality were measured. Plant diet significantly influenced mosquito KD rate at both concentrations of deltamethrin. Following exposure to 0.05% deltamethrin, the *B. lupulina* diet induced a 2.5 fold-increase in mosquito mortality compared to 5% glucose. Species molecular identification confirmed the predominance of *An. gambiae* (60% of the samples) over *An. coluzzii* and *An. arabiensis* in our study area. The *kdr* mutation L1014F displayed an allelic frequency of 0.75 and was positively associated with increased phenotypic resistance to deltamethrin. Plant diet, particularly *B. lupulina,* increased the susceptibility of mosquitoes to insecticides. The finding that *B. lupulina*-fed control individuals (*i.e.* not exposed to deltamethrin) also displayed increased 24 h mortality suggests that plant-mediated effects may be driven by a direct effect of plant diet on mosquito survival rather than indirect effects through interference with insecticide-resistance mechanisms. Thus, some plant species may weaken mosquitoes, making them less vigorous and more vulnerable to the insecticide. There is a need for further investigation, using a wider range of plant species and insecticides, in combination with other relevant environmental factors, to better understand the expression and evolution of insecticide resistance.

## Introduction

Vector management has played an important role in reducing malaria transmission over the past 20 years, and the use of long-lasting insecticidal nets (LLINs) and indoor residual spraying (IRS) have been central components of this control^1^. Pyrethroids, organochlorines, organophosphates and carbamates are the active insecticidal compounds recommended by the World Health Organization (WHO) for IRS, while pyrethroids are the only products used for LLINs^2^. Recently, a mixture of a pyrethroid and a neonicotinoid insecticide for IRS was prequalified by WHO^3^. The massive distribution of mosquito nets over the past 20 years and, perhaps more importantly, the intensive use of insecticides in agricultural practices have led to immense selective pressures for the emergence and spread of insecticide resistance^4–7^. Accordingly, all major malaria vectors have been found to be resistant to the most used classes of insecticides^8^. Important efforts have been previously devoted to understanding the molecular and physiological mechanisms of insecticide resistance as well as its genetic determinants^9–12^. The mechanism, which is best described, involves genetic mutations in the target site of the insecticide that reduce its binding. In particular, the *Ace-1* gene (G119S) mutation confers resistance to organophosphates and carbamates^3^, while mutations in the voltage-gated sodium channel gene (L1014F, L1014S and N1575Y) confer resistance to pyrethroids and organochlorines. The latter mutation is commonly referred to as "knockdown" resistance (*kdr*) because of the ability of individuals with this mutation to not be knocked down following insecticide exposure^9,13,14^.

The majority of ecological studies point to the fact that population exposure to different classes of insecticides is the principal driving factor for the genetic structure of the vector population with regards to mutations conferring resistance^12^. Compared to the important efforts devoted to understand the genetic determinants that modulate the response of malaria vectors to insecticides, little is known about the environmental biotic and abiotic factors that can influence the expression of such tolerance and resistance. Among these environmental factors, one of the most studied is mosquito age, with older individuals being on average less resistant to insecticides than younger counterparts^15–22^. This could be at least partly explained by an age-related decline in the expression of insecticide detoxification genes (*e.g*.^17^). Other studies have also shown that larval nutrition^20,23–25^, adult blood-feeding^26,27^, parasite infection^28–30^, environmental temperature^24,31^, or larval exposure to herbicides and/or metal pollutants^32,33^ can modify adult susceptibility to insecticides.

An important component of mosquito ecology that has been largely overlooked to date concerns the plant feeding of mosquito females. A growing number of studies shows that *Anopheles* females frequently ingest plant fluids as a source of energy and nutrition^34–36^. Sugars, but also amino acids, minerals and vitamins from the fruit, floral and extra-floral nectar, phloem and honeydew can represent an important part of their diet. Despite the biological and epidemiological importance of this behaviour^37–40^, the study of mosquito-plant relationships remains largely neglected. The high degree of plant selection and preference, previously demonstrated in malaria vectors^35,41,42^, has been largely attributed to chemical differences among plant species. In addition, different plants contribute different nutrients and metabolites that can have contrasting impact on mosquito live history traits^34^. A relatively understudied aspect of *Anopheles* ecology, which has implications for control, is how the built-in physiological plasticity to adapt to toxic compounds in their selected plant diet affect their response to insecticides. Indeed, it has been proposed that phytophagy may contribute to the selection of resistance to toxic chemicals in insects^43,44^. Plants often present toxic secondary metabolites against which phytophagous insects have developed resistance mechanisms^45^. In particular, resistance to plant xenobiotics may have formed the basis for the evolution of metabolic resistance to chemical insecticides^44^. However, no study has, to our knowledge, examined the influence of natural plant diet on the susceptibility of *Anopheles* mosquitoes to pyrethroids. In natural conditions, *Anopheles* females can be exposed to a large diversity of plant-related nutrient sources, and it is therefore important to characterize the influence of such environmental variability on their susceptibility to insecticides.

Our study aimed at assessing the effect of plant-related nutrient sources and their combination on the susceptibility of field-derived *An. gambiae *sensu lato (*s.l*.) females to insecticides. Specifically, we evaluated (i) the effect of a range of plant diets on mosquito knockdown rate and 24 h mortality following exposure to either 0.05% or 0.5% of deltamethrin, and (ii) the interaction between plant diet, *kdr* genotypes and insecticide dose on 24 h mosquito mortality.

## Methods

### Mosquito collections

Collections of *An. gambiae s.l.* mosquito larvae were carried out in Diébougou (10°58′N, 3°15′W) in southwestern Burkina Faso. Diébougou is located in the Sudanian climate zone of Burkina Faso where cotton is grown intensively. The average annual rainfall varies from 1000 to 1200 mm during the rainy season, which extends from May to November^46^. Previous studies in this locality showed high level of resistance in *An. gambiae* (*kdr* L1014F mutation frequency of 0.933)^47^. *Anopheles gambiae s.l*. mosquito larvae (3rd and 4th instar) were collected in October and November 2018 using methods described in Service^48^ from natural breeding sites (between 3 and 5 different temporary water pools). Larvae were then pooled and transferred to 1.5 l bottles, and brought back to the insectary. Larvae were fed daily with TetraMin® Baby Fish Food (Tetrawerke, Melle, Germany) under standard controlled conditions (27 ± 2 °C, 70 ± 5% RH and 12 h: 12 h light: dark rhythm) until adult emergence.

### Plant materials and mosquito feeding on plant diet

Two flowering ornamental plants, namely *Barleria lupulina* (*Acanthaceae*) and *Cascabela thevetia* (*Apocynaceae*) were selected as natural sources of nutrient (mostly sugars) for feeding mosquitoes (Supplementary Fig. S1). These plants were selected based on their wide distribution around human dwellings in cities of western Burkina Faso. *Anopheles* readily rest, probe and feed on them^38^. In addition, the ability of these plants to influence mosquito survival and *P. falciparum* transmission potential was previously studied^38^. A solution of 5% glucose (a sugar manufactured by Biosolve Chimie SARL, 57,260 Dieuze, France) was used as a control in our experiments. Collections of *B. lupulina* and *C. thevetia* complied with “Burkina Faso Legislative Assembly” (*Loi 003/2016/AN portant code forestier au Burkina Faso*). These two plants were identified by taxonomist Dr. OUOBA Hermann as in Thiombiano et al*.*^49^, and were deposited in the herbarium of Nazi Boni University, Bobo-Dioulasso, Burkina Faso under ID numbers: UNB-945 and UNB-946, respectively, and kept in the departmental store room. All procedures were conducted in accordance to the relevant institutional, national, and international guidelines and legislation.

The flowers of these plants of unknown age were collected daily between 3 and 4 pm, around human dwellings and public places (gardens, schools) of Bobo-Dioulasso city and surrounding area, and were offered at 5 pm to mosquitoes in 30 cm × 30 cm × 30 cm mesh-covered cages (Supplementary Fig. S1). Plant age was an uncontrolled parameter, but the collected flowers were all open and the wilted or dry flowers were not used. Using scissors, between seven and ten stems of fresh flowers were cut from the plants, arranged in a bouquet and introduced into the mosquito cages (Supplementary Fig. S1). The base of the bouquet was wrapped in moistened paper towels and covered with an aluminum sheet, such that mosquitoes had no access to the moistened paper^38^. Mosquitoes (about 150 individuals from each sex) emerging from field collected larvae were randomly assigned to either *B. lupulina, C. thevetia*, a combination of *B. lupulina* + *C. thevetia*, or the 5% glucose control solution (Supplementary Fig. S1). The flower bouquets, as well as the glucose cotton pads were changed daily at 5 pm. The mosquitoes were allowed to feed on one of these four treatments for three to five days (*i.e.* time deemed sufficient to allow all mosquitoes to feed), after which only the females were aspirated for insecticide susceptibility assays. Here, the exact sources of the nutrients (extra-flower nectar, flower nectar, sap) taken up by mosquito females on plant cuttings (*B. lupulina, C. thevetia* and *B. lupulina* + *C. thevetia*) were not determined. Male and female mosquitoes were given access to their assigned plant treatment until the morning of the test and only the females were aspirated for insecticide susceptibility assays.

### Insecticide susceptibility assays

Three to five day-old *An. gambiae s.l*. females, maintained as described above on one of the four diets (5% glucose*, B. lupulina*, *C. thevetia* or *B. lupulina* + *C. thevetia*) and not blood fed, were used for insecticide susceptibility testing according to the standard WHO tube protocol^2^. Susceptibility testing was performed using deltamethrin, a pyrethroid. These susceptibility assays were carried out using two concentrations of deltamethrin, *i.e.* the 0.05% diagnostic dose recommended by WHO and the 0.5% dose for the determination of resistance intensity^2^. Papers impregnated with deltamethrin (0.05% or 0.5%) and non-impregnated control papers were used for exposing *An. gambiae s.l.* females.

For each diet treatment, between 14 and 31 female mosquitoes (median = 21) were introduced into 6 tubes (4 tubes with insecticide-impregnated papers + 2 control tubes). This experiment was repeated once (*i.e*. two experimental replicates) for each insecticide dose, resulting in a total of 96 tubes and 1696 mosquitoes (6 tubes * 4 plant treatment * 2 deltamethrin doses * 2 experimental replicates). Following mosquito introduction, the presence of KD individuals was recorded at 5, 10, 15, 20, 30, 40, 50 and 60 min. Mosquitoes were then transferred into insecticide-free observation tubes, and maintained on distilled water only. Final mortality in test and control mosquitoes was recorded 24 h after exposure. The tests were conducted under standard bioassay laboratory and insectarium conditions (27 ± 2 °C and 70 ± 5% RH). Following bioassays, species composition of dead and living species after 24 h of exposure, and the resistance gene *kdr* L1014F mutation was determined using PCR as described below.

### Molecular analysis

Genomic DNA was extracted individually from the 1 696 mosquito females by mechanical grinding tissues using the Tissuelyser II (Qiagen) in an extraction buffer (0.1 M Tris HCl, pH 8.0, 0.01 M EDTA, 1.4 M NaCl, 2% cetyl trimethyl ammonium bromide) adapted from Morlais et al*.*^50^. The obtained grinding was incubated at 65 °C for 10 min. Total DNA was extracted with chloroform, precipitated in isopropanol, washed in 70% ethanol and resuspended in sterile water for 12 h. DNA was used to perform PCR to identify *An. gambiae* complex species following the Sine 200X protocol of Santolamazza et al*.*^51^ (Supplementary Information), by using the two primers S200X 6.1F and S200X 6.1R. An additional PCR assay was performed on the same genomic DNA to detect the *kdr* L1014F mutation of the voltage-gated sodium channel (Vgsc) involved in insecticide resistance according to the protocol described by Martinez-Torres et al*.*^13^ using four primers of Kdr_W_Primer_D1, D2, D3, D4 (Supplementary Information), so that each female was characterized for its *kdr* genotype: homozygote sensitive (SS), heterozygote (RS) or homozygote resistant (RR).

### Statistical analyses

All statistical analyses were performed using R software (version 4.0.5)^52^. For each of the two doses (0.5% and 0.05% deltamethrin), a logistic regression by generalized linear model (GLM, binomial errors, or quasibinomial errors) was used to analyze the effect of the diet treatment (4 levels: 5% glucose, *B. lupulina*, *C. thevetia*, and the combination of *B. lupulina* + *C. thevetia*), time (numeric: between 5 and 60 min after exposure), and their two-way interaction, and replicate (2 levels: replicate 1 and replicate 2) as a main effect only on the KD rate of insecticide-exposed mosquitoes. For mosquitoes exposed to 0.05% deltamethrin, predictions were made using the “predict” function to determine the time it would take for mosquitoes to reach 50% KD. For each deltamethrin dose, a binomial GLM was used to determine the effect of the diet treatment, exposure to insecticide (2 levels: control, 0.05% or 0.5% deltamethrin), and their two-way interaction, and replicate as a main effect only on the mortality rate of mosquitoes 24 h following insecticide exposure. Finally, for each insecticide dose, GLMs with binomial or quasi-binomial errors were used to test the effect of plant diet, *kdr* resistance genotypes (SS, RS, RR) and their interaction on the mortality rate of mosquitoes in the deltamethrin-exposed group, and in the control group (mosquitoes not exposed to deltamethrin).

Model simplification used stepwise removal of terms, followed by likelihood ratio replicates (LRT). Term removals that significantly reduced explanatory power (P < 0.05) were retained in the minimal adequate model^53^. Post-hoc tests were used to compare each level of the plant treatment when the latter was significant (‘emmeans’ function of the ‘emmeans’ library, R package version 1.5.5-1)^54^.

### Ethical approval

All procedures were conducted in accordance to the relevant institutional, national, and international guidelines and legislation. Ethical approval was obtained from the Centre Muraz Institutional Ethics Committee (A003-2012/CE-CM) and National Ethics Committee of Burkina Faso (2014–0040).

## Results

### Knockdown rate

#### Deltamethrin (0.05%)

**Figure 1:**
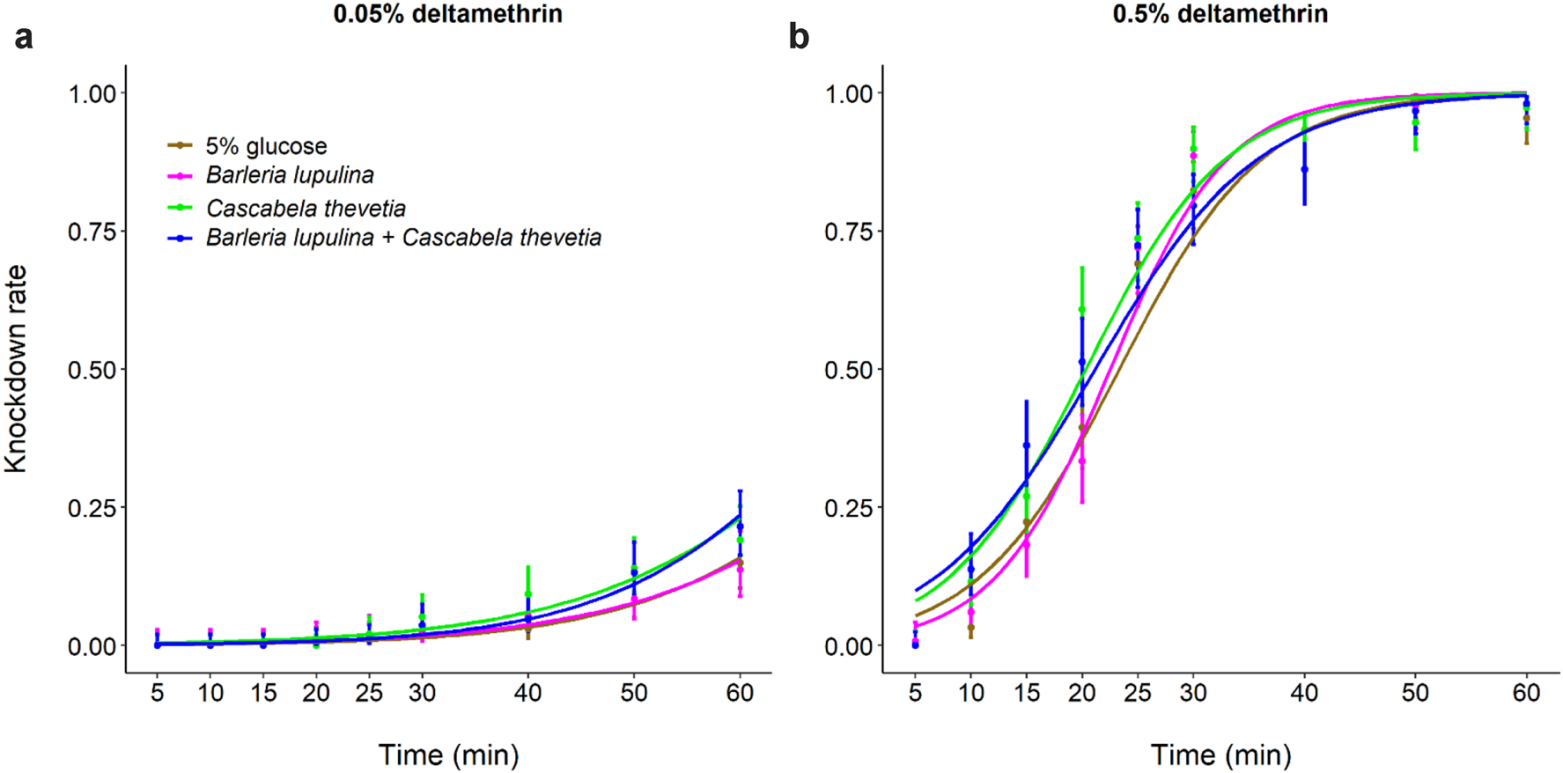
Effect of plant diet on mosquito knockdown rate (KD) following exposure to 0.05% deltamethrin (**a**) or 0.5% deltamethrin (**b**) over time for the two replicates. The lines represent best-fit logistic growth curves for each plant treatment.

Regardless of the plant diet, the overall KD rate was low with only 0.18 ± 0.03 of KD mosquitoes after 60 min (predicted value KD_50_ = 77 min ± 3.42). There was a significant effect of plant diet on KD rate (LRT *X*^*2*^_3_ = 11.63, P = 0.01, Fig. 1a and Supplementary Table S1). In particular, mosquitoes fed with *C. thevetia* had shorter predicted value KD_50_ (74 min ± 3.43) compared to mosquitoes fed with the control, 5% glucose (predicted value KD_50_ = 89 min ± 9.74) (Z = 2.86, P = 0.02, Fig. 1a and Supplementary Table S1). The difference between the treatments was greatest at 60 min of exposure, with mosquitoes fed on *B. lupulina* + *C. thevetia* (KD rate = 0.22 ± 0.06) having approximately twice the KD rate of mosquitoes fed with *B. lupulina* (KD rate = 0.13 ± 0.06) or 5% glucose (KD rate = 0.16 ± 0.06). Furthermore, the KD rate increased with time (LRT *X*^*2*^_1_ = 471.12, P < 0.001, Fig. 1a and Supplementary Fig. S2a) and varied between the two replicates (LRT *X*^*2*^_1_ = 84.71, P < 0.001, Supplementary Fig. S2a), with approximately 30% KD at 60 min for replicate 1 versus only 10% at 60 min for replicate 2 (Supplementary Fig. S2a). Finally, there was no interaction between plant diet and time (LRT *X*^*2*^_3_ = 1.69, P = 0.64, Supplementary Table S1).

#### Deltamethrin (0.5%)

Plant diet significantly influenced mosquito KD (LRT *X*^*2*^_3_ = 25.38, P < 0.001, Fig. 1b). In particular, considering the full observation period, mosquitoes fed on 5% glucose had longer predicted value KD_50_ (24 min ± 1.62) than those fed on *C. thevetia* alone (predicted value KD_50_ = 21 min ± 2.5), Z = 5.12, P < 0.001, Supplementary Table S2) or on *B. lupulina* + *C. thevetia* (23 min ± 3.4, Z = 3.54, P = 0.002, Fig. 1b, Supplementary Table S2). There was an increase of KD over time (LRT *X*^*2*^_1_ = 1024.69, P < 0.001, Fig. 1b and Supplementary Fig. S2b), and replicate effect on KD (LRT *X*^*2*^_1_ = 407.34, P < 0.001, Supplementary Fig. S2b). There was also a significant plant diet by time interaction on KD (LRT *X*^*2*^_3_ = 21.74, P < 0.001, Fig. 1b and Supplementary Fig. S2b), suggesting that the treatment influenced the pace at which mosquitoes got KD following insecticide exposure.

### Mortality rate of mosquitoes 24 h post-exposure

#### Deltamethrin (0.05%)

**Figure 2:**
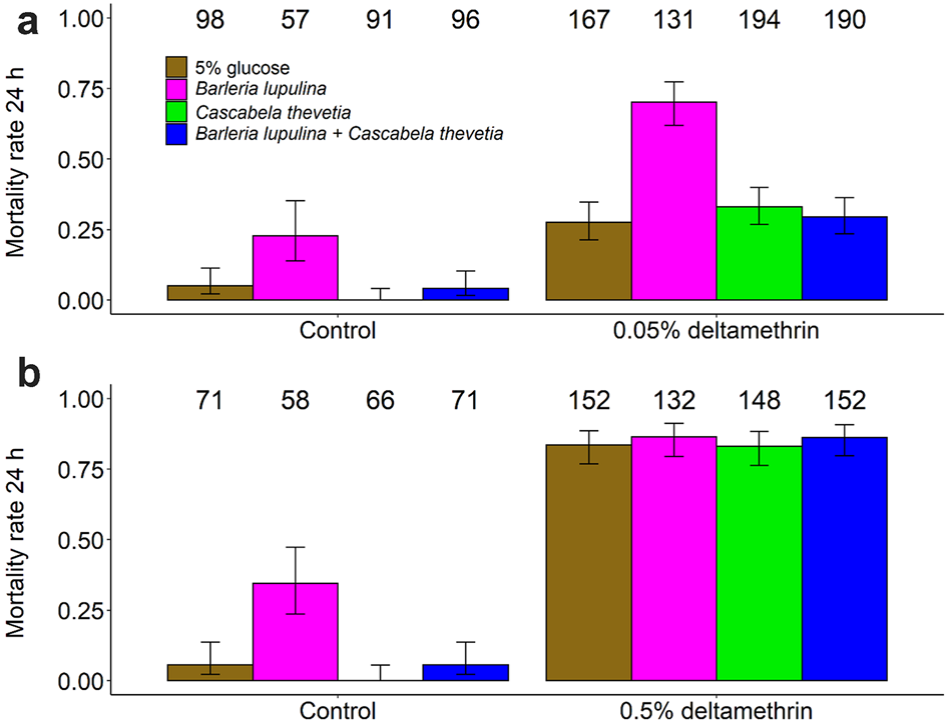
Effect of plant diet and exposure to insecticide (insecticide-exposed vs. unexposed controls) on the mosquito mortality 24 h post-exposure to 0.05% deltamethrin (**a**) or 0.5% deltamethrin (**b**) over 2 replicates. The numbers above the barplots represent the sample size for each plant diet. The error bars represent the variability of data with 95% confidence interval (± 95% CI).

Although 0.05% deltamethrin was not fully efficacious in killing mosquitoes, it significantly decreased their survival with an overall rate of mortality of 0.38 ± 0.04 compared to 0.06 ± 0.03 in controls (LRT *X*^*2*^_1_ = 43.51, P < 0.001, Fig. 2a and Supplementary Table S3, 4). There was a significant effect of plant diet on the mortality of mosquitoes (LRT *X*^*2*^_3_ = 28.03, P < 0.001, Fig. 2a, and Supplementary Table S3), with individuals fed on *B. lupulina* showing higher mortality than those fed on glucose, *C. thevetia*, or the combination *B. lupulina* + *C.* *thevetia*, regardless of the exposure to insecticide (*i.e*. no plant diet by insecticide exposure interaction, Supplementary Fig. S3a and Supplementary Table S3). There was no effect of replicate on mortality of mosquitoes (LRT *X*^*2*^_1_ = 1.18, P = 0.28, Supplementary Fig. S3a).

#### Deltamethrin (0.5%)

There was a highly significant effect of 0.5% deltamethrin on the mortality of female mosquitoes (LRT *X*^*2*^_1_ = 146.27, P < 0.001, Fig. 2b, Supplementary Fig. S3b and Supplementary Table S3, 4), with a 0.85 ± 0.03 mortality in insecticide-exposed individuals compared to 0.11 ± 0.04 in the controls (Supplementary Table S4). Plant diet influenced mosquito mortality (LRT *X*^*2*^_3_ = 54.64, P < 0.001, Fig. 2b, Supplementary Fig. S3b), but this effect was mainly driven by increased mortality in *B. lupulina*-fed and control mosquitoes. Accordingly, there was a significant interaction between plant diet and insecticide exposure (LRT *X*^*2*^_3_ = 34.07, P < 0.001, Fig. 2b and Supplementary Fig. S3b). Finally, there was a significantly effect of replicate on the mosquito mortality (LRT *X*^*2*^_1_ = 26.32, P < 0.001, Supplementary Fig. S3b).

### Effect of plant diet, kdr genotype and their interactions on the mosquito mortality

Among the field-derived females used in this experiment*, An. gambiae* (n = 1 021) was predominant, followed by *An. coluzzii* (n = 313) and *An. arabiensis* (n = 307) (Supplementary Table S5). Molecular analysis detected the *kdr* L10114F mutation in these three species at high frequency (*i.e.* 0.9, 0.6 and 0.51 in *An. gambiae, An. coluzzii* and *An. arabiensis,* respectively). Genotypic frequencies are given in Supplementary Table S5. The following analysis includes all *Anopheles gambiae s.l.* mosquitoes, and separate analysis for each of the three species are displayed in Supplementary Table S6 and Supplementary Fig. S5.

#### Deltamethrin (0.05%)

**Figure 3:**
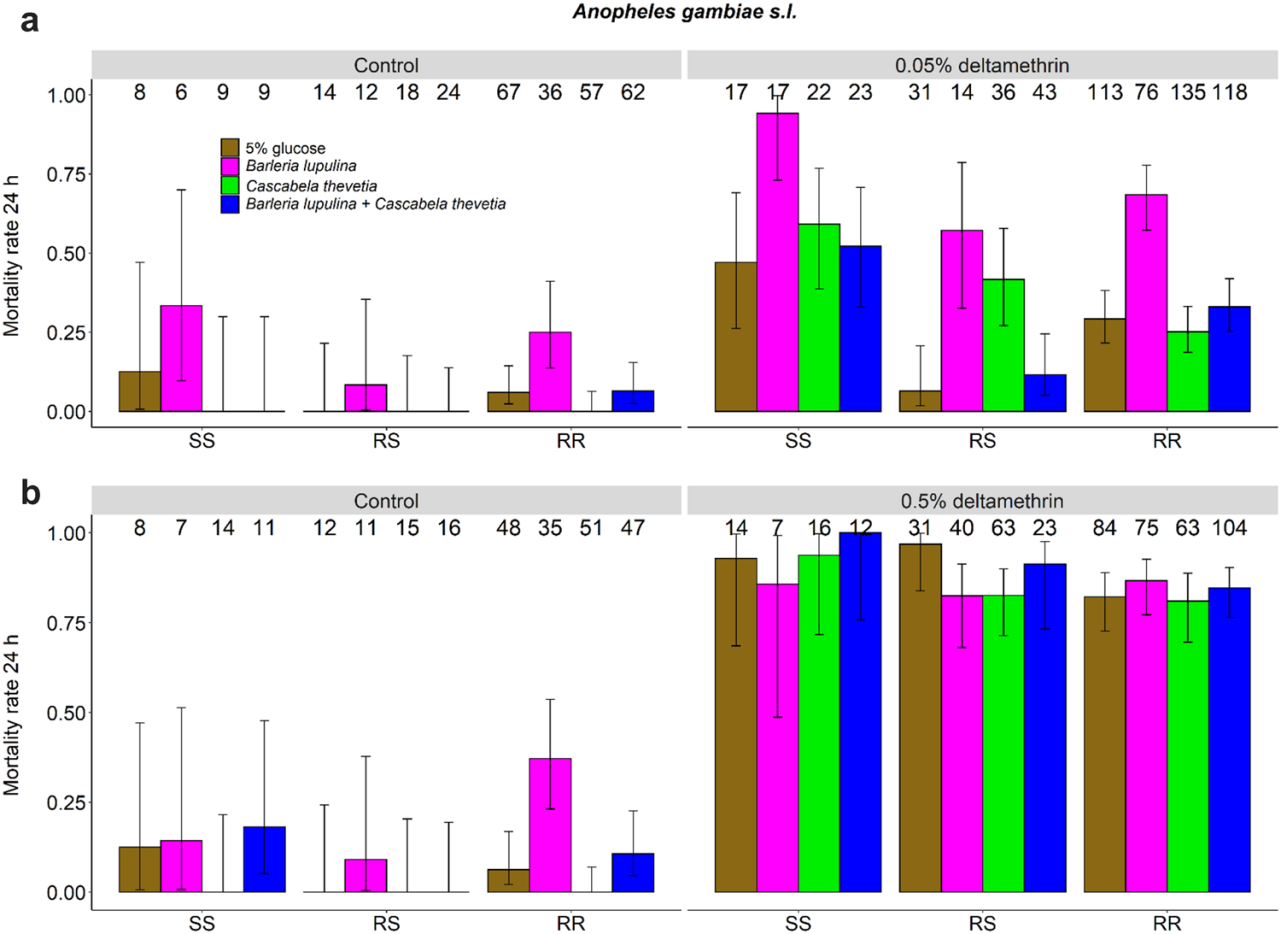
Effect of insecticide exposure, plant diet and *kdr* resistance genotype on the proportion of dead mosquitoes exposed to (**a**) 0.05% deltamethin, or (**b**) 0.5% deltamethrin over two replicates. The numbers above the barplots represent the sample size for each plant treatment. The letters on the x-axis correspond to the different *kdr* genotypes with SS designating homozygous susceptible mosquitoes, RS heterozygous mosquitoes and RR homozygous resistant mosquitoes. The error bars represent the variability of data with 95% confidence interval (± 95% CI).

In the presence of a low dose of the insecticide, the *kdr* L1014F mutation conferred a two- to three- fold reduction in mortality (SS = 0.62 ± 0.14, RS = 0.24 ± 0.15, RR = 0.36 ± 0.07, LRT *X*^*2*^_2_ = 11.80; P = 0.003, Fig. 3a, multiple pairwise comparisons: SS > RS, Z = 4.63, P = 0.001, SS > RR, Z = 3.66, P = 0.001 and RR > RS, Z = 2.43, P = 0.04). There was a significant interaction between *kdr* genotype and plant diet (LRT *X*^*2*^_6_ = 17.38; P = 0.01), indicating that the effect of plant treatment on mosquito mortality varied among the genotypes.

In the absence of the insecticide, the effect of the *kdr* genotype on the mosquito mortality was marginally non-significant (SS = 0.09 ± 0.33, RS = 0.01 ± 0.24, RR = 0.08 ± 0.13, LRT *X*^*2*^_2_ = 5.33; P = 0.07, Fig. 3a), and there was no significant interaction between *kdr* genotype and plant diet (Fig. 3a, LRT *X*^*2*^_6_ = 2.54; P = 0.86).

#### Deltamethrin (0.5%)

When exposed to a high insecticide dose, the effect of the *kdr* genotype on the mosquito mortality was marginally non-significant, with a mortality of 0.94 ± 0.07, 0.87 ± 0.06 and 0.84 ± 0.04 for SS, RS and RR genotypes, respectively (LRT *X*^*2*^_2_ = 5.06; P = 0.08, Fig. 3b). The interaction between *kdr* genotype and plant diet was not significant (LRT *X*^*2*^_6_ = 7.08; P = 0.31, Fig. 3b).

In the absence of the insecticide, the effect of the *kdr* genotype on the mosquito mortality was moderate (SS = 0.10 ± 0.29, RS = 0.02 ± 0.26 and RR = 0.12 ± 0.14, LRT *X*^*2*^_2_ = 7.30; P = 0.03, Fig. 3b), and no significant pairwise differences were found on the basis of multiple comparison tests. Finally, there was no significant interaction between *kdr* genotype and plant diet (LRT *X*^*2*^_6_ = 3.23; P = 0.78, Fig. 3b).

## Discussion

The objective of this study was to assess the effect of natural plant diet on the response of *An. gambiae s.l*. to insecticides. Our findings showed that the effect of plant diet on mosquito resistance, as measured by KD rate and 24 h mortality, is significant. In particular, plant treatment influenced mosquito KD rate at both the 0.05% WHO diagnostic dose of deltamethrin and the 0.5% dose. Plant treatment also induced a varying level of mortality in both mosquitoes exposed to deltamethrin 0.05% and unexposed control individuals. At the higher dose of deltamethrin, mosquito mortality became high, regardless of the plant species received, while the effect of the plants was still apparent in the unexposed controls.

Overall, less than 25% of the adult females were KD one hour following exposure to 0.05%, 0.05% deltamethrin, and it took about 25 min to induce 50% KD with the higher dose of 0.5%. Besides, about 40% and 85% of the individuals were recovered dead 24 h after exposure to 0.05% and 0.5% deltamethrin, respectively. This means that the mosquitoes were highly resistant according to the WHO criteria, *i.e.* a mortality less than 90% at the discriminating concentration of 0.05% (1×) and less than 98% at the 10 × dose^2^. These results confirm the high insecticide resistance status of this mosquito population^55^. Deltamethrin is a pyrethroid that targets the voltage-gated sodium channel in insect neurons, and mutations in this target site are a likely cause of insecticide resistance^12^. Accordingly, our genotyping revealed a high frequency of the L1014F *kdr* point mutation (90% in *An. gambiae,* the predominant species in our samples), and our SS genotypes displayed a 2- to threefold increase in mortality at 24 h following exposure to 0.05% deltamethrin compared to the RS and RR genotypes (Fig. 3a). As this study was performed on mosquitoes collected at the larval stage in the field, we cannot exclude the possibility that they have been exposed, during part of their development in their natural breeding sites, to environmental stressors, such as pollutants or herbicides, which are known to improve subsequent adult resistance to insecticides^32,33^. It would be meaningful to repeat this experiment using susceptible and resistant mosquitoes with the same genetic background, and reared under the same conditions (food, density, etc.).

Our results showed a strong effect of plant diet on mosquito insecticide resistance, with some plant species conferring greater or lesser susceptibility to mosquitoes. In particular, the average mortality rates at 24 h following exposure to 0.05% deltamethrin was 2.5 fold greater in mosquitoes that were fed on a *B. lupulina* diet (70%) relative to the control (27.5%) (Fig. 2a and Supplementary Table S4). One obvious implication is that the intensity of insecticide resistance in natural conditions will vary spatially and temporally depending on fluctuations in the diversity of plant nutrient sources. For example, areas where mosquitoes get a significant portion of their meals from plant species such as *B. lupulina* would display decreased insecticide resistance. That being said, such simple predictions may prove wrong as different genetic and environmental parameters (age, larval nutrition, female body size, temperature, infection, etc.) and their combinations (*i.e.* G x E interactions) may have either opposite, additive, or synergistic effects on the expression of insecticide resistance.

The precise mechanism of plant-mediated effects on mosquito resistance is not yet clear, but at least four non-mutually exclusive hypothetical processes can be proposed. First, it is possible that plant diet interferes with some of the well-known molecular mechanisms of resistance, which improve mosquito survival upon insecticide contact (*e.g.* enzyme-mediated detoxification, such as cytochrome P450s). For instance, *B. lupulina* might decrease enzyme activities resulting in reduced resistance to deltamethrin, and hence in increased mosquito mortality. In contrast, plants can contain vitamins, such as ascorbic acid (present in nectars but in fruits especially), which have important antioxidant properties^56^. In this scenario, mosquito feeding on vitamin-rich plant diet could reduce the oxidative stress induced by exposure to insecticides and increase resistance to insecticides. It has previously been demonstrated that dietary antioxidants, such as vitamin C can improve DDT phenotypic resistance in *Drosophila melanogaster*^57^, and it would be important to corroborate these findings in mosquitoes.

Second, differences in resistance among plant treatments could have been the result of uneven distribution of *kdr* genotypes among the different plant treatments. Mosquitoes were maintained for 3–5 days on their plant diet before the bioassay. Thus, if some genotypes survive better on some plants than others, then selection can occur during this pre-test period resulting in an uneven distribution of the different genotypes among the treatments. For instance, if the SS genotype survive well on *B. lupulina* (and conversely the RR and RS genotypes survive poorly), this genotype would therefore become overrepresented in this treatment, leading to the observed lower resistance. This mechanism can, however, be partly ruled out as the *kdr* genotype composition was similar across the different plant treatments (Supplementary Table S7).

Third, plant diet can have an indirect effect on insecticide resistance through mosquito physiological condition and energetic status. Both field and laboratory based studies, have shown that plant diet can affect mosquito life history traits, such as survival^34,37^. The current study demonstrates that plant diet can also modulate the 24 h mortality in both control and deltamethrin-exposed adults. The effect of mosquito plant diet on delayed mortality after insecticide exposure may be related to the amount of nutritional reserves available, as suggested by studies on the effects of larval nutrition on adult insecticide resistance (*e.g.*^24^). Plant diet may indeed differ in their nutritional quality and in turn affect mosquito general vigor. In particular, mosquitoes that fed on *B. lupulina* would accumulate less energy reserves, making them less resistant to the insecticide. In addition, mosquitoes might display varying preferences for plants. For example, mosquitoes exposed to *B. lupulina* might be less likely to feed and therefore have lower energy reserves compared to other plant diets for which mosquitoes would be fully replete before the insecticide test. Beyond plant nutritional quality and mosquito energy level, there could also be direct toxic effects of *B. lupulina* on mosquitoes, which may ultimately weaken mosquitoes and render them more susceptible to insecticides. Although not characterized in this study, the presence of toxic chemicals in these natural plants could temporarily affect the metabolism of mosquitoes, thus modulating their tolerance to insecticides. *Barleria lupulina* contains secondary metabolites, such as terpenes, flavonoids, lignins, alkaloids, particularly the iridoid glycosides, and most plant products containing terpenes are efficient insecticides^58^.

Fourth, plant diet could change the microbiota composition of mosquito females^59,60^, which in turn could modify mosquito response to deltamethrin^61–64^. Our results showed that mosquito KD rate and mortality varied among the two replicates. This could also be explained by mosquito microbiota, as females used in replicate 1 and 2 came from different breeding sites and hatching batches^60^.

Mosquito natural 24 h mortality varied with plant diet. Unexposed control mosquitoes fed on *B. lupulina* displayed increased mortality (Fig. 2), above the standard set by WHO^65^. This result suggests that plant-mediated effects on mosquito mortality following exposure to insecticide may in fact be driven by a direct plant effect on mosquito survival rather than interference with insecticide resistance per se. The ability to distinguish between these two possibilities is one of the advantages of using insecticide-unexposed control mosquitoes. In the vast majority of bioassays evaluating the effects of environmental factors (*e.g.* mosquito age, larval nutrition and infection) on the phenotypic expression of mosquito resistance to insecticides, unexposed controls are lacking. These unexposed controls, consisting in individuals introduced into WHO tubes with solvent-impregnated papers, also suffer some level of stress, and may reveal a difference in 24 h mortality among treatments, *e.g.* mosquito age group. Therefore, the need to investigate, as part of insecticide bioassays, the direct effect of environmental factors on mosquito life-history traits in using unexposed control individuals is strongly advocated.

Although the *kdr* genotypes (RR, RS and SS) seemed to perform equally well on the plant treatments during the 3–5 days pre-test, leading to an even genotype distribution among treatments (Supplementary Table S7), our results showed a significant interaction between *kdr* genotype and plant diet on mosquito 24 h mortality following exposure to 0.05% deltamethrin (Fig. 3a). This significant interaction indicates that the effect of plant diet on mosquito pyrethroid response depends on the mosquito *kdr* genotype. For example, glucose-fed and 0.05% deltamethrin-exposed RS mosquitoes displayed very low mortality compared to glucose-fed SS and RR (Fig. 3a). A key evolutionary consequence of the influence of plant diversity on insecticide resistance is the maintenance of genetic diversity at the *kdr* locus in *Anopheles* populations. Indeed, some genotypes may be sensitive to constituents present in one plant species, while other genotypes would perform better when feeding on other plant species (*i.e.* G x E interactions). This, in addition to the possible existence of a cost of this resistance, may partly explain the maintenance of polymorphism at the *kdr* gene and why the R allele is not completely fixed in areas of high insecticide coverage.

Plant-related variations in susceptibility to deltamethrin were not consistent for both knockdown and mortality rates, *i.e.* plant species that increased KD were not systematically the same that increased 24 h mortality (Fig. 1, 2). Interestingly, the relationship between KD and 24 h mortality varied among the plant treatments (Supplementary Fig. S4): there was a positive relationship between KD and 24 h mortality in the 5% glucose, *C. thevetia* and the combination *B. lupulina* + *C. thevetia* treatment, but no such link was found for *B. lupulina* (Supplementary Fig. S4). This supports the observation that *B. lupulina* induced both the lowest KD and the highest 24 h mortality, and suggests that KD and 24 h mortality, the two response variables classically measured as part of WHO tube test, do not share the same underlying proximate mechanisms, and that it may depend on the plant diet considered. Specifically, it is conceivable that *B. lupulina* compounds would induce a slow toxic effect, at first resulting in a low KD following exposure to deltamethrin. Subsequently, after continued metabolism, plant toxicity would be concomitant with the toxic effect of deltamethrin, causing the high mortality observed at 24 h. Although our results only partially confirm the previous finding that KD cannot be used as a reliable indicator of 24-h mortality^66^, they emphasize that this relationship may be plant diet-dependent.

## Conclusion & perspectives

This study provides proof of principle that natural plant diet can affect phenotypic expression of pyrethroid resistance in *Anopheles* mosquitoes. The underlying mechanisms are still unknown but the use of control individuals not exposed to the insecticide suggests that some plant species exploited as nutrients sources may weaken mosquitoes, making them more vulnerable to the insecticide in terms of induced 24 h mortality. Beyond immediate KD and mortality, future studies should explore the impact of both plant diet and insecticide exposure on a wider range of mosquito life-history traits and on long term survival in particular. Since KD rate seem an unreliable predictor of 24 h mortality, using dose–response assays^66^ to evaluate the effect of plant diet and insecticide exposure on diverse longer-term mosquito life history traits is recommended. It would also be interesting to use mosquito males to confirm the results obtained here with females. Only two plant species and their combination were used in this study and there is a need for further investigation into the influence of natural vegetal nutrient sources on insecticide toxicity using a wider range of plant species in combinations with other relevant factors (*i.e.* E x E interactions), considering local availability and phenology. For example, it would be particularly interesting to explore how plant diet affects the expression of resistance in mosquitoes of varying ages. Examining how plant diet can affect other resistance mechanisms, such as metabolic resistance, would also be a valuable perspective. Finally, our findings emphasize that considering plant species used by malaria vectors for food is important to better understand the ecology and evolution of insecticide resistance, which may in turn affect disease transmission dynamics and vector control management.

## Supplementary Information

Supplementary Figures.

Supplementary Tables.

## Data Availability

The dataset is available from the corresponding author upon reasonable request.

## References

[1] Bhatt S (2015). The effect of malaria control on *Plasmodium falciparum* in Africa between 2000 and 2015. Nature.

[2] WHO. Test procedures for insecticide resistance monitoring in malaria vector mosquitoes (2016).

[3] WHO. Guidelines for malaria vector control (2019). 30844152

[4] Gnankiné O (2013). Insecticide resistance in *Bemisia tabaci* Gennadius (Homoptera: Aleyrodidae) and *Anopheles gambiae* Giles (Diptera: Culicidae) could compromise the sustainability of malaria vector control strategies in West Africa. Acta Trop..

[5] Ranson H, Lissenden N (2016). Insecticide resistance in African *Anopheles* mosquitoes: A worsening situation that needs urgent action to maintain malaria control. Trends Parasitol..

[6] Reid MC, McKenzie FE (2016). The contribution of agricultural insecticide use to increasing insecticide resistance in African malaria vectors. Malar. J..

[7] Huijben S, Paaijmans KP (2017). Putting evolution in elimination: Winning our ongoing battle with evolving malaria mosquitoes and parasites. Spec. Issue Rev. Synth..

[8] WHO. Global technical strategy for malaria 2016–2030, 2021 update (2021).

[9] Ranson H (2000). Identification of a point mutation in the voltage-gated sodium channel gene of Kenyan *Anopheles gambiae* associated with resistance to DDT and pyrethroids. Insect Mol. Biol..

[10] Weill M (2004). The unique mutation in *ace-1* giving high insecticide resistance is easily detectable in mosquito vectors. Insect Mol. Biol..

[11] Ranson H (2011). Pyrethroid resistance in African anopheline mosquitoes: What are the implications for malaria control?. Trends Parasitol..

[12] Hemingway J, Hawkes NJ, McCarroll L, Ranson H (2004). The molecular basis of insecticide resistance in mosquitoes. Insect Biochem. Mol. Biol..

[13] Martinez-Torres D (1998). Molecular characterization of pyrethroid knockdown resistance (kdr) in the major malaria vector *Anopheles gambiae s.s*.. Insect Mol. Biol..

[14] Jones C (2012). Footprints of positive selection associated with a mutation (*N1575Y*) in the voltage-gated sodium channel of *Anopheles gambiae*. Proc. Natl. Acad. Sci. U. S. A..

[15] Hunt RH, Brooke BD, Pillay C, Koekemoer LL, Coetzee M (2005). Laboratory selection for and characteristics of pyrethroid resistance in the malaria vector *Anopheles funestus*. Med. Vet. Entomol..

[16] Glunt KD, Thomas MB, Read AF (2011). The effects of age, exposure history and malaria infection on the susceptibility of *Anopheles* mosquitoes to low concentrations of pyrethroid. PLoS One.

[17] Rajatileka S, Burhani J, Ranson H (2011). Mosquito age and susceptibility to insecticides. Trans. R. Soc. Trop. Med. Hyg..

[18] Chouaibou MS (2012). Increase in susceptibility to insecticides with aging of wild *Anopheles gambiae* mosquitoes from Côte d’Ivoire. BMC Infect. Dis..

[19] Jones CM (2012). Aging partially restores the efficacy of malaria vector control in insecticide-resistant populations of *Anopheles gambiae s.l.* from Burkina Faso. Malar. J..

[20] Kulma K, Saddler A, Koella JC (2013). Effects of age and larval nutrition on phenotypic expression of insecticide-resistance in *Anopheles* Mosquitoes. PLoS ONE.

[21] Aïzoun N, Aïkpon R, Azondekon R, Asidi A, Akogbéto M (2014). Comparative susceptibility to permethrin of two *Anopheles gambiae s.l*. populations from Southern Benin, regarding mosquito sex, physiological status and mosquito age. Asian Pac. J. Trop. Biomed..

[22] Collins E (2019). The relationship between insecticide resistance, mosquito age and malaria prevalence in *Anopheles gambiae s.l*. from Guinea. Sci. Rep..

[23] Oliver S, Brooke B (2013). The effect of larval nutritional deprivation on the life history and DDT resistance phenotype in laboratory strains of the malaria vector *Anopheles arabiensis*. Malar. J..

[24] Owusu HF, Chitnis N, Müller P (2017). Insecticide susceptibility of *Anopheles* mosquitoes changes in response to variations in the larval environment. Sci. Rep..

[25] Sovegnon, P. M., Fanou, M. J., Akoton, R. & Djihinto, O. Y. Effects of larval diet on the life-history traits and phenotypic expression of pyrethroid resistance in the major malaria vector *Anopheles gambiae s.s. *Preprint at *bioRxiv* http://doi.org/10.1101/2022.01.11.475801 (2022).

[26] Halliday WR, Feyereisen R (1987). Why does DDT toxicity change after a blood meal in adult female *Culex pipiens*?. Pestic. Biochem. Physiol..

[27] Oliver SV, Lyons CL, Brooke BD (2022). The effect of blood feeding on insecticide resistance intensity and adult longevity in the major malaria vector *Anopheles funestus* (Diptera: Culicidae). Sci. Rep..

[28] Farenhorst M (2009). Fungal infection counters insecticide resistance in African malaria mosquitoes. Proc. Natl. Acad. Sci. U. S. A..

[29] Koella JC, Saddler A, Karacs TPS (2012). Blocking the evolution of insecticide-resistant malaria vectors with a microsporidian. Evol. Appl..

[30] Alout H (2014). Interplay between *Plasmodium* infection and resistance to insecticides in vector mosquitoes. J. Infect. Dis..

[31] Glunt KD, Oliver SV, Hunt RH, Paaijmans KP (2018). The impact of temperature on insecticide toxicity against the malaria vectors *Anopheles arabiensis* and *Anopheles funestus*. Malar. J..

[32] Oliver S, Brooke B (2018). The effect of commercial herbicide exposure on the life history and insecticide resistance phenotypes of the major malaria vector *Anopheles arabiensis* (Diptera: culicidae). Acta Trop..

[33] Oliver S, Brooke B (2018). The effect of metal pollution on the life history and insecticide resistance phenotype of the major malaria vector *Anopheles arabiensis* (Diptera: Culicidae). PLoS ONE.

[34] Foster WA (1995). Mosquito sugar feeding and reproductive energetics. Annu. Rev. Entomol..

[35] Nyasembe VO, Tchouassi DP, Pirk CWW, Sole CL, Torto B (2018). Host plant forensics and olfactory-based detection in Afro-tropical mosquito disease vectors. PLoS Negl. Trop. Dis..

[36] Barredo E, DeGennaro M (2020). Not just from blood: Mosquito nutrient acquisition from nectar sources. Trends Parasitol..

[37] Stone CM, Foster WA, Takken W, Koenraadt C (2013). Plant-sugar feeding and vectorial capacity. Ecology of Parasite-Vector Interactions.

[38] Hien DFDS (2016). Plant-mediated effects on mosquito capacity to transmit human malaria. PLoS Pathog..

[39] Stone C, Witt A, Walsh G, Foster W, Murphy S (2018). Would the control of invasive alien plants reduce malaria transmission? A review. Parasites Vectors.

[40] Ebrahimi B (2018). Alteration of plant species assemblages can decrease the transmission potential of malaria mosquitoes. J. Appl. Ecol..

[41] Manda H (2007). Discriminative feeding behaviour of *Anopheles gambiae s.s*. on endemic plants in western Kenya. Med. Vet. Entomol..

[42] Nyasembe VO (2014). *Plasmodium falciparum* infection increases *Anopheles gambiae* attraction to nectar sources and sugar uptake. Curr. Biol..

[43] Després L, David JP, Gallet C (2007). The evolutionary ecology of insect resistance to plant chemicals. Trends Ecol. Evol..

[44] Nkya TE, Akhouayri I, Kisinza W, David JP (2013). Impact of environment on mosquito response to pyrethroid insecticides: Facts, evidences and prospects. Insect Biochem. Mol. Biol..

[45] Li X, Schuler MA, Berenbaum MR (2007). Molecular mechanisms of metabolic resistance to synthetic and natural xenobiotics. Annu. Rev. Entomol..

[46] Bationo CS (2021). Spatio-temporal analysis and prediction of malaria cases using remote sensing meteorological data in Diébougou health district, Burkina Faso, 2016–2017. Sci. Rep..

[47] Namountougou M (2013). First report of the L1014S *kdr* mutation in wild populations of *Anopheles gambiae* M and S molecular forms in Burkina Faso (West Africa). Acta Trop..

[48] Service MW (1977). A critical review of procedures for sampling populations of adult mosquitoes. Bull. Entomol. Res..

[49] Thiombiano, A. *et al.* Catalogue des plantes vasculaires du Burkina Faso. In *Boissiera* Vol. 65 (ed Cyrille Chatelain) (Conservatoire et Jardin botaniques, 2012).

[50] Morlais I, Ponçon N, Simard F, Cohuet A, Fontenille D (2004). Intraspecific nucleotide variation in *Anopheles gambiae*: New insights into the biology of malaria vectors. Am. J. Trop. Med. Hyg..

[51] Santolamazza F (2008). Insertion polymorphisms of *SINE200* retrotransposons within speciation islands of *Anopheles gambiae* molecular forms. Malar. J..

[52] R Core Team. A language and environment for statistical computing (2021).

[53] Crawley MJ (2007). The R Book.

[54] Lenth, R. V. emmeans: Estimated marginal means, aka least-squares means (2021).

[55] Hien A (2021). Evidence supporting deployment of next generation insecticide treated nets in Burkina Faso: Bioassays with either chlorfenapyr or piperonyl butoxide increase mortality of pyrethroid-resistant *Anopheles gambiae*. Malar. J..

[56] Nicolson SW, Nepi M, Pacini E (2007). Nectaries and Nectar.

[57] Abdu-Allah G (2020). Dietary antioxidants impact DDT resistance in *Drosophila melanogaster*. PLoS ONE.

[58] Gnankiné O, Bassolé ILHN (2017). Essential oils as an alternative to pyrethroids’ resistance against *Anopheles* species complex giles (Diptera: Culicidae). Molecules.

[59] Gendrin, M. & Christophides, G. K. The *Anopheles* mosquito microbiota and their impact on pathogen transmission. In *Anopheles Mosquitoes—New Insights into Malar. Vectors* (ed. Manguin, S.) (IntechOpen, 2013).

[60] Saab SA (2020). The environment and species affect gut bacteria composition in laboratory co-cultured *Anopheles gambiae* and *Aedes albopictus* mosquitoes. Sci. Rep..

[61] Dada N, Sheth M, Liebman K, Pinto J, Lenhart A (2018). Whole metagenome sequencing reveals links between mosquito microbiota and insecticide resistance in malaria vectors. Sci. Rep..

[62] Barnard K, Jeanrenaud ACSN, Brooke BD, Oliver SV (2019). The contribution of gut bacteria to insecticide resistance and the life histories of the major malaria vector *Anopheles arabiensis* (Diptera: Culicidae). Sci. Rep..

[63] Omoke D (2021). Western Kenyan *Anopheles gambiae* showing intense permethrin resistance harbour distinct microbiota. Malar. J..

[64] Pelloquin B (2021). Overabundance of *Asaia* and *Serratia* Bacteria is associated with deltamethrin insecticide susceptibility in *Anopheles coluzzii* from Agboville, Côte d’Ivoire. Microbiol. Spectr..

[65] WHO. Test procedures for insecticide resistance monitoring in malaria vector mosquitoes (2013).

[66] Owusu HF, Jančáryová D, Malone D, Müller P (2015). Comparability between insecticide resistance bioassays for mosquito vectors: Time to review current methodology?. Parasites Vectors.

