# Supplementary material for: Natural plant diet impacts phenotypic expression of pyrethroid resistance in Anopheles mosquitoes
Source: Sci Rep. 2022 Dec 12;12:21431. doi: 10.1038/s41598-022-25681-6 (PMC9744732; doi:10.1038/s41598-022-25681-6)
Supplement: Supplementary file 1 — Supplementary Figures. [file 41598_2022_25681_MOESM1_ESM.docx]

**Supplementary file**


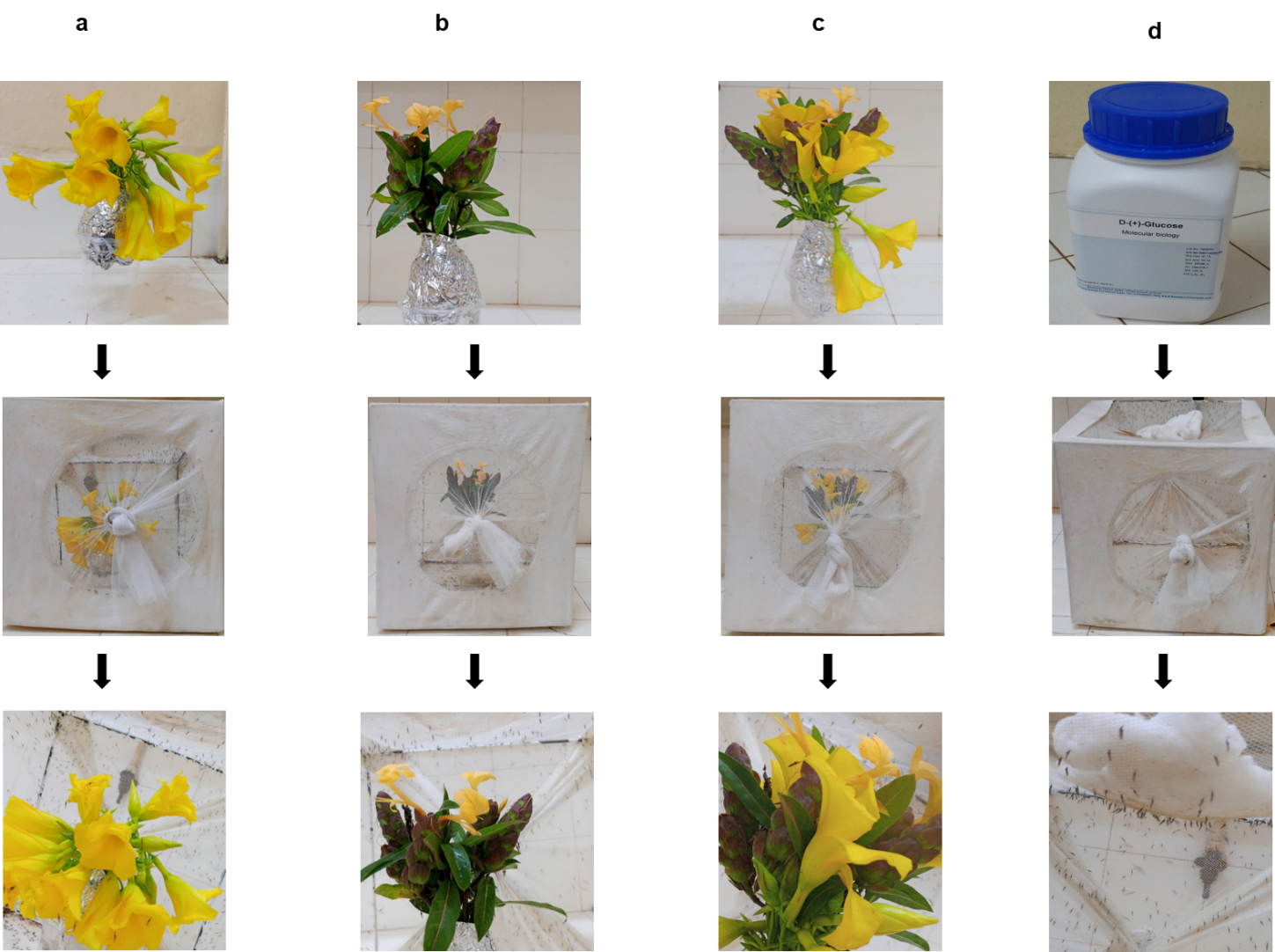


**Supplementary Figure S1**: Experimental setup of mosquito plant feeding experiment. Bundle of flowers of *Cascabela thevetia* (a), *Barleria lupulina* (b), *Barleria lupulina + Cascabela thevetia* (c) and, 5% glucose solution on cotton pad (d) and their introduction into the cages for mosquito feeding.

**Complete list of primers**

The primers used for the detection of the SINE 200X 6.1 gene locus were S200X 6.1F: TCG-CCT-TAG-ACC-TTG-CGT-TA; S200X 6.1R: CGC-TTC-AAG-AAT-TCG-AGA-TAC. The four primers of Kdr_W_Primer were D1: ATA-GAT-TCC-CCG-ACC-ATG; D2: AGA-CAA-GGA-TGA-TGA-ACC; D3: AAT-TTG-CAT-TAC-TTA-CGA-CA and D4: CTG-TAG-TGA-TAG-GAA-ATT-TA.

***
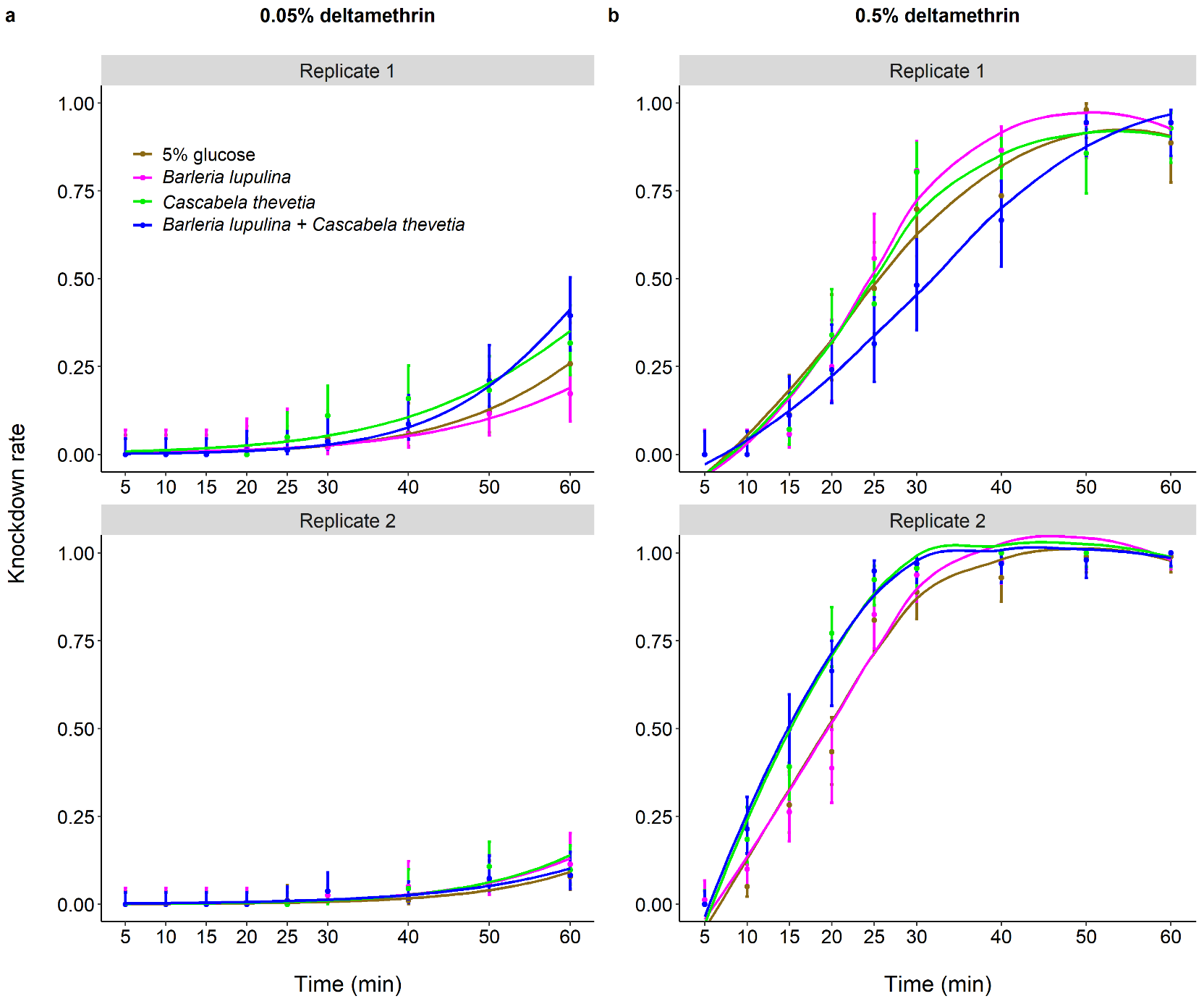
***

**Supplementary Figure S2**: Effect of plant diet on mosquito knock down rate (KD) following exposure to 0.05% deltamethrin (a) or 0.5% deltamethrin (b) over time for each replicate. The lines represent best-fit logistic growth curves for each plant treatment.

**
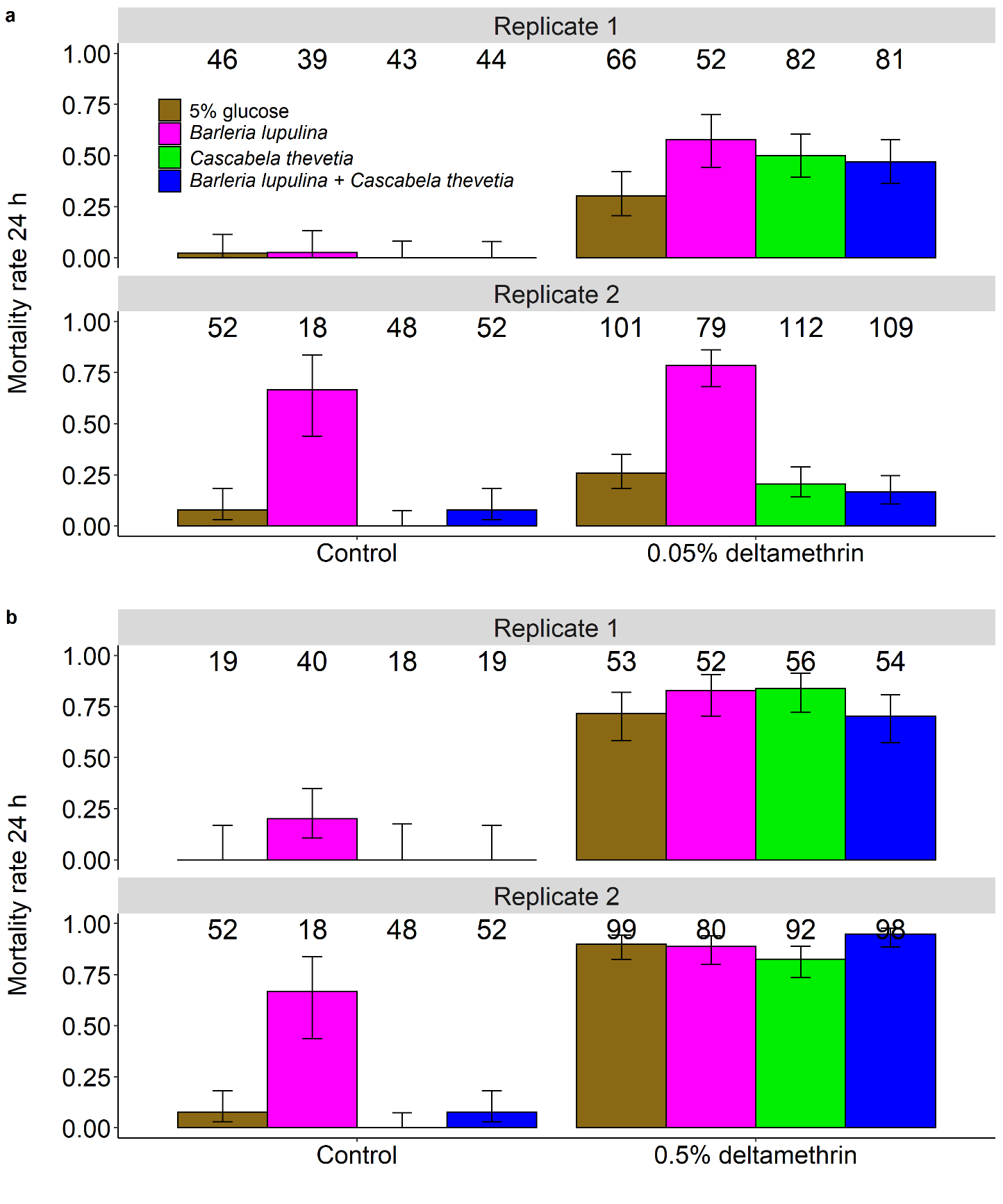
Supplementary Figure S3:** Effect of plant diet and insecticide dose on the proportion of dead mosquitoes after 24 h of exposure to (a) 0.05% deltamethrin (a) and to (b) 0.5% deltamethrin for each replicate. The numbers above the barplots represent the sample size of each level of the plant diet variable. The error bars represent the variability of data with 95% confidence interval (±95% CI).

**
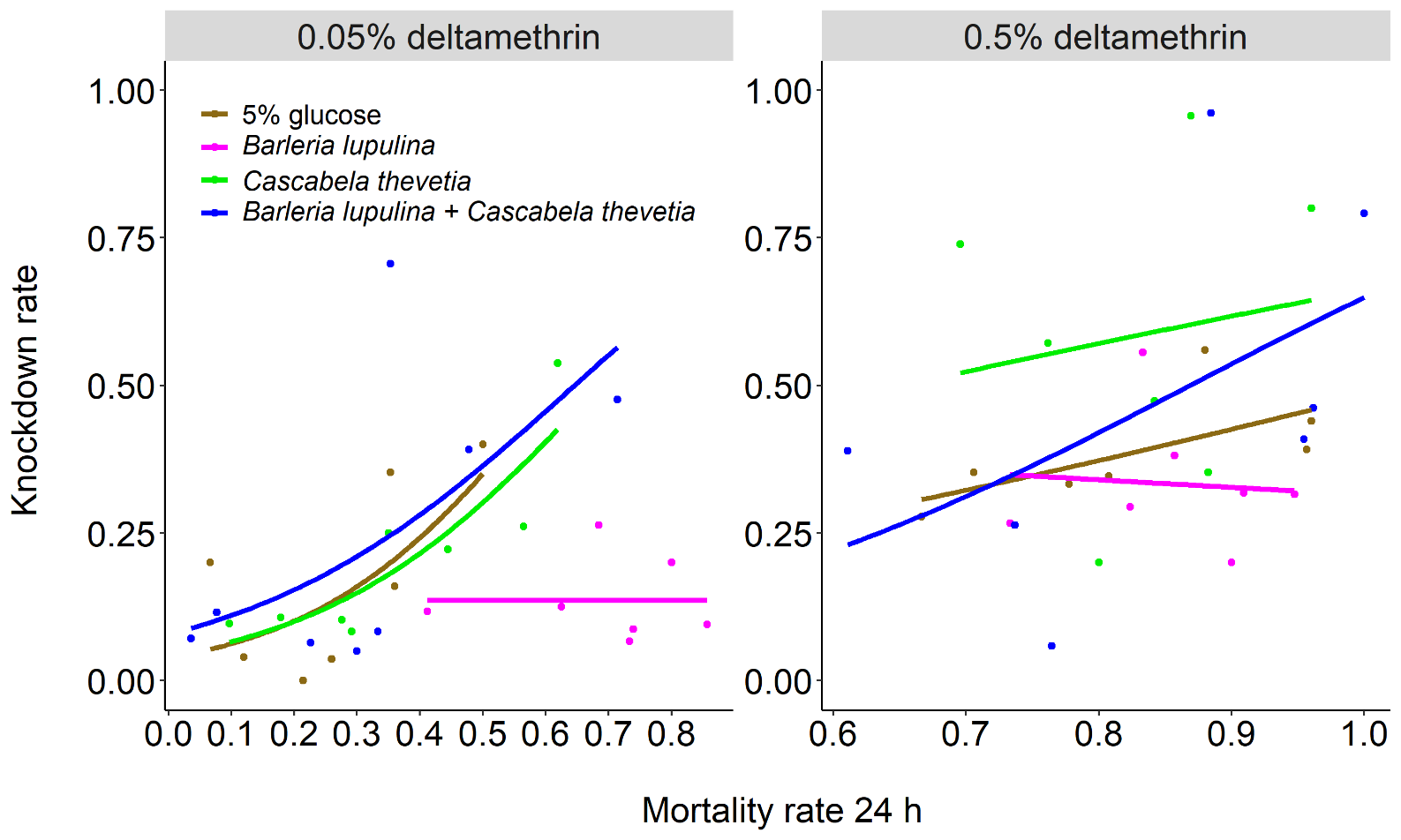
**

**Supplementary Figure S4:** Relationship between proportion of KD and 24 h mosquito mortality. (a) KD 60 min with the 0.05% deltamethrin and (b) KD 20 min with the 0.5% deltamethrin. The lines represent best-fit logistic growth curves for each plant treatment and the dots represent the number of WHO tubes used in the bioassays.

**
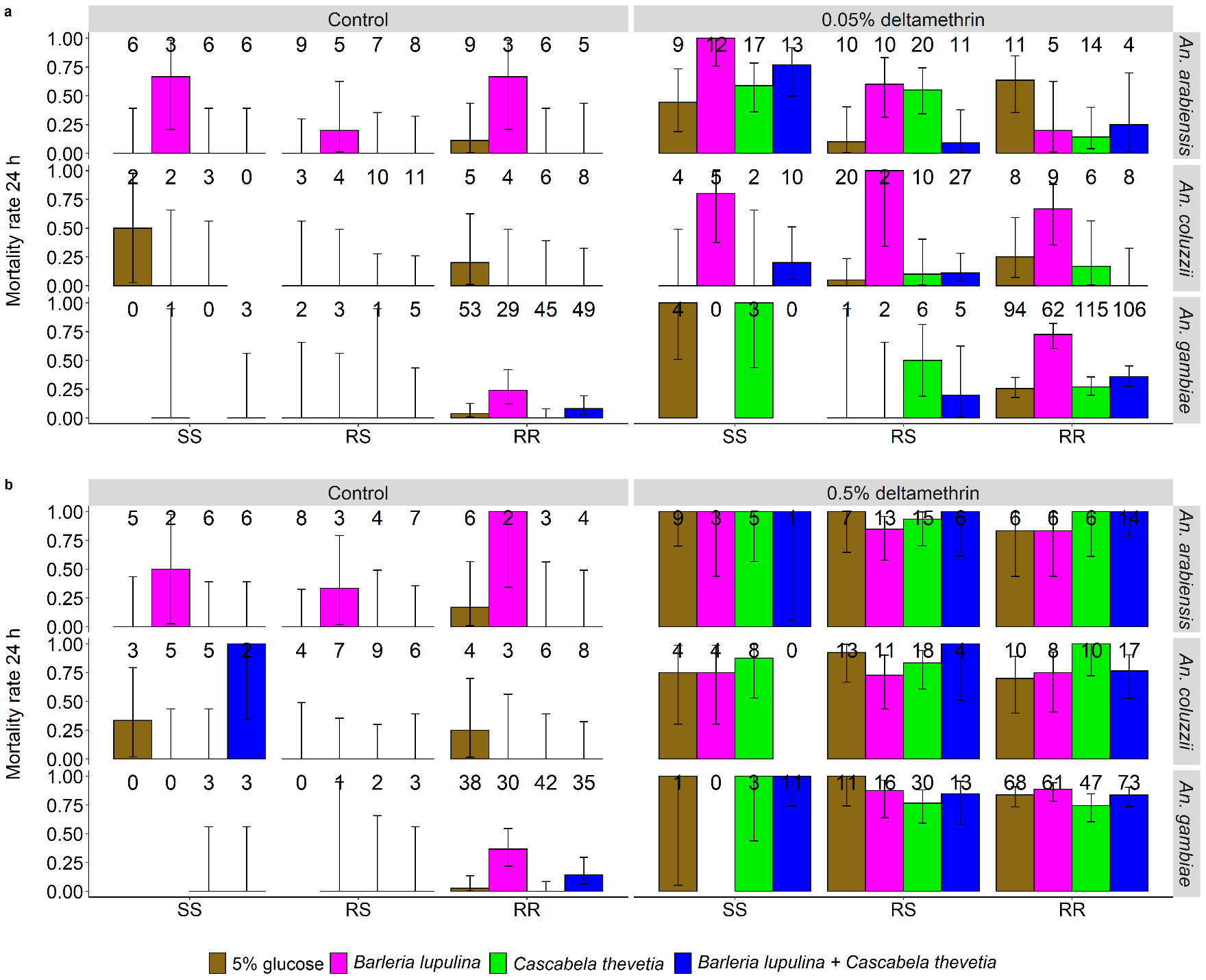
**

**Supplementary Figure S5:** Effect of insecticide exposure, mosquito species, plant diet, and *kdr* resistance gene on the proportion of dead mosquitoes exposed to (a) 0.05% deltamethin, or (b) 0.5% deltamethrin over two replicates. The numbers above the barplots represent the sample size for each plant treatment. The error bars represent the variability of data with 95% confidence interval (±95% CI). The letters on the x-axis correspond to the different genotypes of the *kdr* resistance gene with SS designating homozygous susceptible mosquitoes, RS heterozygous mosquitoes and RR homozygous resistant mosquitoes.
