# Supplementary material for: Natural plant diet impacts phenotypic expression of pyrethroid resistance in Anopheles mosquitoes
Source: Sci Rep. 2022 Dec 12;12:21431. doi: 10.1038/s41598-022-25681-6 (PMC9744732; doi:10.1038/s41598-022-25681-6)
Supplement: Supplementary file 2 — Supplementary Tables. [file 41598_2022_25681_MOESM2_ESM.docx]

**Supplementary file**

**Supplementary Table S1:** Statistical analyses of the effect of plant diet on the knockdown of *Anopheles gambiae* *s.l*. exposed to deltamethrin

|  | **0.05% deltamethrin** | | | **0.5% deltamethrin** | | |
| --- | --- | --- | --- | --- | --- | --- |
| Explanatory variables | LRT *X^2^* | df | P | LRT *X^2^* | df | P |
| Plant diet | 11.63 | 3 | **0.01** | 25.38 | 3 | **< 0.001** |
| Time | 471.12 | 1 | < **0.001** | 1024.69 | 1 | **< 0.001** |
| Replicate | 84.71 | 1 | < **0.001** | 407.34 | 1 | < **0.001** |
| Plant diet: Time | 1.69 | 3 | 0.64 | 21.74 | 3 | **< 0.001** |

LRT = Likelihood Ratio

*χ^2^* = Chisq square

df = degree of freedom

P = P-value

**Supplementary Table S2:** Knockdown rate over time for each plant diet and each dose of deltamethrin (replicates 1 and 2 combined)

| **Insecticide** | **Mosquito** | **Plant diet** | **% KD (min) ± 95 CI** | | | | | | | | | |
| --- | --- | --- | --- | --- | --- | --- | --- | --- | --- | --- | --- | --- |
|  |  |  | 5 | 10 | 15 | 20 | 25 | 30 | 40 | 50 | 60 | 5-60 |
| 0.05% deltamethrin | *An. gambiae s.l.* | 5% glucose | 0  0  0  0 | 0  0  0  0 | 0  0  0  0 | 0.01±0.01  0.01±0.01  0  0.01±0.01 | 0.01±0.02  0.02±0.02  0.02±0.02  0.01±0.01 | 0.02±0.02  0.02±0.03  0.05±0.03  0.04±0.03 | 0.03±0.03  0.05±0.04  0.09±0.04  0.05±0.03 | 0.08±0.04  0.08±0.05  0.14±0.05  0.13±0.05 | 0.15±0.05  0.14±0.06  0.20±0.06  0.22±0.06 | 0.03±0.01  0.04±0.01  0.06±0.01  0.05±0.01 |
|  |  | *B. lupulina* |  |  |  |  |  |  |  |  |  |  |
|  |  | *C. thevetia* |  |  |  |  |  |  |  |  |  |  |
|  |  | *B. l+C. T* |  |  |  |  |  |  |  |  |  |  |
| 0.5% deltamethrin | *An. gambiae s.l*. | 5% glucose | 0  0.01±0.01  0  0 | 0.03±0.03  0.06±0.04  0.12±0.05  0.14±0.05 | 0.22±0.07  0.18±0.07  0.27±0.07  0.36±0.08 | 0.40±0.08  0.33±0.08  0.61±0.08  0.51±0.08 | 0.69±0.07  0.72±0.08  0.74±0.07  0.72±0.07 | 0.82±0.06  0.89±0.05  0.90±0.05  0.80±0.06 | 0.86±0.05  0.93±0.04  0.93±0.04  0.862±0.05 | 0.99±0.02  0.98±0.03  0.95±0.04  0.97±0.03 | 0.95±0.03  0.98±0.03  0.97±0.03  0.98±0.02 | 0.55±0.03  0.56±0.03  0.61±0.03  0.59±0.03 |
|  |  | *B. lupulina* |  |  |  |  |  |  |  |  |  |  |
|  |  | *C. thevetia* |  |  |  |  |  |  |  |  |  |  |
|  |  | *B. l+C. T* |  |  |  |  |  |  |  |  |  |  |

*B. l+C. t*: Combination of *B. lupulina + C. thevetia*

**Supplementary Table S3**: Statistical analyses of the effect of plant diet on the mortality of *Anopheles gambiae* *s.l.* 24 h following exposure to deltamethrin

|  | **0.05% deltamethrin** | | | **0.5% deltamethrin** | | |
| --- | --- | --- | --- | --- | --- | --- |
| Explanatory variables | LRT *X^2^* | df | P | LRT *X^2^* | df | P |
| Plant diet | 28.03 | 3 | **< 0.001** | 54.64 | 3 | **< 0.001** |
| Insecticide | 43.51 | 1 | **< 0.001** | 146.27 | 1 | **< 0.001** |
| Replicate | 1.18 | 1 | 0.28 | 26.32 | 1 | < **0.001** |
| Plant diet: insecticide | 2.69 | 3 | 0.44 | 34.07 | 3 | **<0.001** |

**Supplementary Table S4**: Mortality rate (±95CI) 24 h following exposure for each plant diet and each dose of deltamethrin

| **Insecticide/Control** | **5% glucose** | ***B. lupulina*** | ***C. thevetia*** | ***B. lupulina + C. thevetia*** | **Insecticide (all plant diet)** |
| --- | --- | --- | --- | --- | --- |
| 0.05% deltamethrin | 0.275±0.0678 | 0.702±0.0783 | 0.330±0.0662 | 0.295±0.0648 | 0.4±0.04 |
| CTRL | 0.05±0.04 | 0.23±0.11 | 0 | 0.04±0.04 | 0.06±0.03 |
| All | 0.19±0.05 | 0.56±0.07 | 0.23±0.05 | 0.21±0.05 | - |
| 0.5% deltamethrin | 0.84±0.06 | 0.86±0.06 | 0.83±0.06 | 0.86±0.05 | 0.85±0.03 |
| CTRL | 0.06±0.054 | 0.35±0.12 | 0 | 0.06±0.05 | 0.11±0.04 |
| All | 0.59±0.06 | 0.71±0.06 | 0.56±0.07 | 0.61±0.06 | - |

CTRL = Control (paper not impregnated with insecticide)

**Supplementary Table S5**: Genotypic resistance status of *Anopheles gambiae* *s.l.*

| **Species** | **n** | **SS** | **RS** | **RR** | **Undetermined** | **Allelic frequencies** | |
| --- | --- | --- | --- | --- | --- | --- | --- |
|  |  |  |  |  |  | **S** | **R** |
| *An. arabiensis* | 307 | 92(30%) | 121(39%) | 90(29%) | 4(1%) | 0.50 | 0.49 |
| *An. coluzzii* | 313 | 55(18%) | 144(46%) | 111(35%) | 3(1%) | 0.41 | 0.58 |
| *An. gambiae* | 1021 | 30(3%) | 97(10%) | 868(85%) | 26(3%) | 0.08 | 0.90 |
| Undetermined | 55 | 9(16%) | 12(22%) | 15(27%) | 19(35%) | 0.27 | 0.38 |
| **N** | 1696 | 186(11%) | 374(22%) | 1084(64%) | 52(3%) | 0.22 | 0.75 |

N = total sample size, n = sample size for each species, SS = homozygote susceptible genotype, RS = heterozygote genotype, RR = homozygote resistant genotype

**Supplementary Table S6:** Statistical analyses of effect of the plant diet, *kdr* resistance gene and their interaction on the proportion of dead *Anopheles gambiae* *s.l.*

| **Control** | | | **0.05% deltamethrin** | | | **Control** | | | **0.5% deltamethrin** | | |  |  |
| --- | --- | --- | --- | --- | --- | --- | --- | --- | --- | --- | --- | --- | --- |
| LRT *X^2^* | df | P | LRT *X^2^* | ddl | P | LRT *X^2^* | df | P | LRT *X^2^* | df | P |  |  |
| *An. gambiae s.l.* | Plant diet | 26.81 | 3 | **<0.001** | 11.95 | 3 | **0.01** | 33.28 | 3 | **<0.001** | 1.79 | 3 | 0.62 |
|  | *kdr* | 5.33 | 2 | 0.07 | 11.81 | 2 | **0.003** | 7.30 | 2 | **0.03** | 5.06 | 2 | 0.08 |
|  | Plant diet : *kdr* | 2.55 | 6 | 0.86 | 17.38 | 6 | **0.008** | 3.23 | 6 | 0.78 | 7.08 | 6 | 0.31 |
| *An. arabiensis* | Plant diet | 19.90 | 3 | **<0.001** | 12.35 | 3 | **0.01** | 18.98 | 3 | **<0.001** | 4.86 | 3 | 0.18 |
|  | *kdr* | 3.65 | 2 | 0.16 | 7.09 | 2 | **0.03** | 5.40 | 2 | 0.07 | 2.57 | 2 | 0.28 |
|  | Plant diet : *kdr* | 0.90 | 6 | 0.99 | 26.67 | 6 | **<0.001** | 0.00 | 6 | 1 | 2.25 | 6 | 0.90 |
| *An. coluzzii* | Plant diet | 6.19 | 3 | 0.10 | 23.77 | 3 | **<0.001** | 9.48 | 3 | **0.02** | 2.18 | 3 | 0.54 |
|  | *kdr* | 2.23 | 2 | 0.33 | 0.17 | 2 | 0.92 | 8.91 | 2 | **0.01** | 0.31 | 2 | 0.85 |
|  | Plant diet : *kdr* | 0.00 | 5 | 1 | 7.54 | 6 | 0.27 | 5.26 | 6 | 0.51 | 6.58 | 5 | 0.25 |
| *An. gambiae* | Plant diet | 15.96 | 3 | **0.001** | 39.56 | 3 | **<0.001** | 27.41 | 3 | **<0.001** | 5.62 | 3 | 0.13 |
|  | *kdr* | 3.46 | 2 | 0.18 | 18.42 | 2 | **<0.001** | 2.59 | 2 | 0.27 | **6.14** | **2** | **0.05** |
|  | Plant diet : *kdr* | 0.00 | 4 | 1 | 7.08 | 4 | 0.13 | 0.00 | 3 | 1 | 3.13 | 5 | 0.68 |

| **Treatment** | **n** | **SS** | **RS** | **RR** | **Undetermined** | **Allelic frequencies** | |
| --- | --- | --- | --- | --- | --- | --- | --- |
|  |  |  |  |  |  | **S** | **R** |
| 5% glucose | 429 | 45(10%) | 80(19%) | 283(66%) | 21(5%) | 0.20 | 0.75 |
| *B. lupulina* | 339 | 35(10%) | 76(22%) | 213(63%) | 15(4%) | 0.22 | 0.74 |
| *C. thevetia* | 472 | 56(12%) | 124(26%) | 281(60%) | 11(2%) | 0.25 | 0.73 |
| *B. lupulina+C. thevetia* | 456 | 50(11%) | 94(21%) | 307(67%) | 5(1%) | 0.21 | 0.78 |
| **N** | 1696 | 186(11%) | 374(22%) | 1084(64%) | 52(3%) | 0.22 | 0.75 |

**Supplementary Table S7**: Genotypic resistance status according to plant treatment
